# Hypertensive disorders of pregnancy are associated with an inflammatory state: evidence from hematological findings and cytokine levels

**DOI:** 10.1186/s12884-019-2383-7

**Published:** 2019-07-09

**Authors:** Yohana Silas Mtali, Magdalena Amani Lyimo, Lucio Luzzatto, Siriel Nanzia Massawe

**Affiliations:** 1 Department of Hematology and blood transfusion, Muhimbili University of Health and Allied Sciences (MUHAS), Dar es Salaam, Tanzania; 2 Department of Obstetrics and Gynaecology, Muhimbili University of Health and Allied Sciences (MUHAS), Dar es Salaam, Tanzania

**Keywords:** Anemia, Pregnancy, Hypertensive disorders in pregnancy, Inflammation, Cytokines, Platelets, Pre-eclampsia, Eclampsia

## Abstract

**Background:**

Abnormalities of blood cell counts and of cytokine profiles in women with hypertensive disorders of pregnancy (HDP) have been reported in several studies. Although their cause-effect relationships to HDP are not yet clear, detecting and monitoring these alterations can be of use for prognosis and management of HDP. This study aimed to determine hematological, coagulation and cytokine profiles in hypertensive as compared to normotensive pregnancy and to identify correlations between these profiles.

**Methods:**

This was a hospital-based comparative cross-sectional study conducted from September 2017 to February 2018. There were two groups: the comparison group consisted of 77 normotensive pregnant women attending the antenatal clinic of Muhimbili National Hospital (MNH); the index group consisted of 76 hypertensive pregnant women admitted to the maternity block of the same hospital. Hematological and cytokine parameters were compared between the hypertensive and the normotensive group. We analyzed the data using Student’s independent t-test when the data were normally distributed; and the Mann–Whitney U-test when the data were not normally distributed. Kruskal Wallis with Dunn’s multiple comparison tests was run for subgroup analysis and correlation studies were done using Spearman ranking.

**Results:**

Hemoglobin levels were slightly but significantly lower, (*P* < 0.01) in women with HDP compared to normotensive (N) women; the same was true for platelet counts (*P* < 0.001). The red cell distribution width (RDW) was slightly but significantly higher in HDP than in N. Neutrophil counts and Interleukin 6 (IL-6) levels were significantly (*P* < 0.001) higher in HDP than in N; and within HDP IL-6 levels increased with increasing severity of HDP. A novel remarkable finding was that eosinophil counts, normal in N, were lower and lower with increasing severity of HDP, to the point that they were nearly absent in women with eclampsia.

**Conclusion:**

There are significant changes in hematological, cytokine and coagulation parameters in pregnant women with hypertensive disorders compared to normotensive pregnant women. The picture that emerges is that of an inflammatory state associated with hypertensive disorders of pregnancy.

## Background

Hypertensive disorders of pregnancy (HDP) affect up to 10% of all pregnancies globally [1]. These multi-system disorders comprise gestational hypertension (GH), pre-eclampsia (PE) and eclampsia (E). A majority of HDP cases are from low and middle income countries [2]; in Tanzania at Muhimbili national hospital the prevalence is 5.1% [3].

Hypertensive disorders of pregnancy are not only a common cause of morbidity and mortality in pregnant women: they can also affect the fetus and the newborn. Indeed, complications of HDP include premature delivery, intrauterine growth retardation (IUGR), *abruptio placentae* and intrauterine death [4]. Maternal complications include the HELLP syndrome (hemolyisis, elevated liver enzymes, low platelets), pulmonary edema, acute liver/renal failure, disseminated intravascular coagulopathy, adult respiratory distress syndrome, sepsis and liver hemorrhage [2].

Despite a vast literature on these disorders, the pathophysiology of HDP is not fully elucidated. One common thread is inflammation [5]. With respect to developing HDP, the following risk factors are known: previous pre-eclampsia or hypertension in pregnancy, chronic kidney disease, hypertension, diabetes (type 1 or type 2), and autoimmune disorders (including systemic lupus erythematous or anti phospholipids syndrome) [6]. Moreover, first pregnancy, age > 40 years, > 10 year interval from last pregnancy, body-mass index(BMI) > 35 kg/m^2^, polycystic ovarian syndrome, history of pre-eclampsia in the family and multiple pregnancies are considered to be moderate risk factors [7].

Several studies have shown that the hematological, cytokine and coagulation profiles in pregnant women with HDP differ from those in normotensive pregnant women. Specifically, studies have found that IL-6 levels, TNF-α levels, neutrophil to lymphocyte ratios and platelet count may predict disease development, and may help in monitoring disease and in prognosis of HDP [8–14].

There is considerable variation in lifetime risk of HDP and in mortality rates from HDP in different populations [15]. HDP is a highly complex syndrome where multiple genetic, nutritional and other environmental factors intervene; at the same time, hematologic and cytokine parameters may vary not only in relation to gender and age, but also in different populations [16]. Therefore it would not be justified to extrapolate to our Sub-Saharan population the findings from Asian, European or American studies: rather, we decided to investigate important hematological, coagulation and cytokine laboratory features of HDP in Tanzanian women.

## Methods

### Study and design, setting

This was a hospital-based comparative cross-sectional study conducted from September 2017 to February 2018. There were two groups: the comparison group consisted of 77 normotensive pregnant women attending the antenatal clinic of Muhimbili National Hospital (MNH); the index group consisted of 76 hypertensive pregnant women with confirmed diagnosis of gestational hypertension (GH) (*n* = 14), preeclampsia (PE) (*n* = 44), or eclampsia (E)(*n* = 18) who were admitted to the maternity block of the same hospital.

Muhimbili national hospital (MNH) in Tanzania caters to a population of over five million Dar es Salaam residents (according to NBS 2017 data) and neighboring coastal regions. MNH also serve as a teaching hospital for Muhimbili University of Health and Allied Sciences (MUHAS). It has a special pre-eclampsia/eclampsia ward with a capacity of 15-beds, to which patients with HDP are admitted. The samples were analyzed at central pathology laboratory (CPL)-MNH which is ISO 15189 accredited.

### Recruitment of study subjects, case definition and sampling

The patients with HDP were either from the MNH ante natal clinic or referred from other hospitals within the city of Dar-s-Salaam. When hypertension was observed the patients may have been started on low dose aspirin and this was continued in the HDP clinic. All those who, during the study period, met the inclusion criteria were included without any selection. Blood samples from all patients were taken as soon as they were admitted and always before delivery.

The normotensive pregnant women were recruited during visits at the MNH antenatal clinic that is attended mostly by women with normal pregnancy that has health insurance cover. Blood samples were obtained, after they had consented to participate in the study, whenever they were due for a routine blood test (e.g. Hb level); of course this was always before delivery.

The sample size was calculated using Info stat Calculator version 7 (sample size calculation for unmatched cohort/cross-sectional study) whereby 95% two sided confidence interval was used, with the 80% power of study. Ratio of normotensive to HDP to cases was 1:1, with the proportion of outcome in HDP and normotensive group being 29 and 7% respectively whereby a minimum of 112 (56 index and 56 comparative) was obtained.

Gestational hypertension was defined as raised blood pressure (> 140/90 mmHg) measured 6 h apart occurring for the first time at ≥20 weeks of pregnancy but without proteinuria (< 300 mg/24 h). Preeclampsia was defined as the presence of hypertension (blood pressure > 140/90 mmHg) measured 6 h apart associated with proteinuria (> 300 mg/24 h) in women known to be previously normotensive occurring at ≥20 weeks of pregnancy [1]. Eclampsia was defined as the occurrence of convulsions and/or coma unrelated to other cerebral conditions in women with signs and symptoms of PE [1].

The Exclusion criteria included having fever at time of blood collection, having history of blood transfusion in the current pregnancy, having pre-existing chronic diseases (heart failure, hypertension, diabetes or renal failure).

### Procedures and data collection

Participants’ data on socio-demographic characteristics, blood transfusion history, parity, blood pressure, gestational age, duration from last pregnancy and number of missed pregnancies were collected. Data were obtained through structured questionnaires and/or by extracting information from clinic cards and from the hospital files.

About 4.5 ml of venous blood were drawn into a 5 ml vacutainer tube containing 0.5 ml of tri-potassium ethylene-di-amine tetra acetic acid (K3EDTA). Full blood count was done using Abbott Cell dyne 3700.

The blood sample for prothrombin time (PT) and partial thromboplastin time (PTT) was collected into a vacuum tube (blue cap) containing sodium citrate (32.06 mg/mL, final concentration 3.8%) in a 9∶1 volume ratio. Platelet poor plasma (PPP) was prepared by double centrifugation at 2500 *g* for 15 min. Coagulation tests were done using the Sysmex CA 500 machine. Quality controls for both machines were done before the sample analysis.

Plasma levels of IL6, IL 10 and TNF- α were determined from EDTA plasma using enzyme-linked immunoassay assay (ELISA) on ELx 800 plate reader at 450 absorbance. ELISA reagents were from Invitrogen laboratories, Biocenter2/1030 Vienna Austria. Sensitivity of IL-6, IL-10 and TNF - α kits was < 1 pg/mL, < 3 pg/mL and < 1.7 pg/mL with assay range of 10.24–400 pg/mL, 15.36–600 pg/mL and 15.6–1000 pg/mL respectively.

### Data analysis

The data were analyzed using SPSS version 23; Continuous variables were checked for normality. Differences in hematological, coagulation and cytokine parameters were compared between the HDP and normotensive groups using Student’s independent t-test and Mann–Whitney U-test when the data were normally and abnormally distributed respectively.

Kruskal Wallis with Dunn’s multiple comparison tests was also run for subgroup analysis comparing the hematological parameters in both HDP and normotensive groups. Spearman ranking correlation was used to correlate the inflammatory cytokines with hematological parameters. A *p* value of < 0.05 was considered to be statistically significant. Some of the parameters in this study were also compared to data from non-pregnant women established in our population by previous study of Saathoff et al. in 2008 [17].

## Results

### Demographic, socio-economic and clinical characteristics of study participants

A total of 153 women were recruited to this study. The median age was 29 years. More than a third of participants had only primary education or below; two thirds were either formally employed or entrepreneurs. The median gestational age was 33 weeks. Majority (78%) of study participants had no history of miscarriage or fetal loss (Table 1).

**Table 1:** Demographic, socio-economic and clinical characteristics of study participants

		Pregnant women		
Variable	All	Normotensive	Hypertensive	*P* value
Overall		*N* = 77	*N* = 76	
Median age (SD)	29 (6.6)	30.8 (4.9)	28.5 (6.1)	0.015
Education	*N* = 152			< 0.001
Primary and below n (%)	59	13 (22)	46 (78)	
Secondary n (%)	40	17 (42.5)	23 (57.5)	
College n (%)	53	46 (86.8)	7 (13.2)	
Marital status	*N* = 143			0.04
Married n (%)	143	77 (53.8)	66 (46.2)	
Employment	*N* = 153			< 0.001
Formal work n (%)	54	40 (74.1)	14 (25.9)	
Self-employed n (%)	54	25 (46.3)	29 (53.7)	
No formal work n (%)	45	11 (24.4)	34 (75.6)	
History of miscarriage/fetal loss	N = 153			0.305
None n (%)	120	57 (47.5)	63 (52.5)	
>/= 1 n (%)	33	19 (57.6)	14 (42.4)	
Gravid status	N = 152			
First pregnancy n (%)	53	25 (47.2)	28 (52.8)	
2nd pregnancy and above n (%)	99	54 (51.5)	46 (48.5)	0.61
Median Gestational age (IQR)	33.0 (29.0, 36.0)	33 (28,36)	34 (30,35)	0.346
Median systolic blood pressure (IQR)	120.0 (110.0, 132.8)	111 (108,120)	132 (120,140)	< 0.001
Median diastolic blood pressure (IQR)	70.0 (64.0, 89.0)	66 (62,70)	86 (70,91)	< 0.001

The median gestational ages were similar in both groups. Median diastolic and systolic pressure were significantly higher – by definition – in the HDP group than in the normotensive group. Women with HDP were slightly younger than those without HDP (*P* = 0.015). A significantly higher proportion of normotensive women had a college education, and were either formally employed or self-employed (*P* = 0.001) (Table 1).

### Hematological and cytokine parameters in the normotensive group and in the hypertensive group

**Fig. 1:**
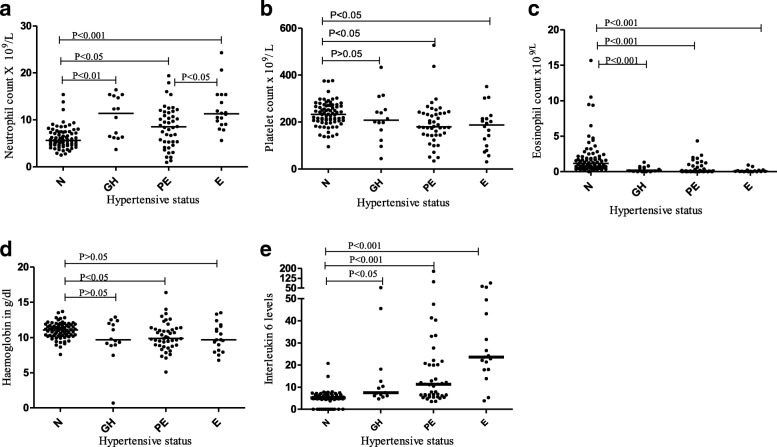
Distributions of selected hematological and cytokines parameters. In each panel N means normotensive, GH means gestational hypertension, PE means preeclampsia and E means eclampsia. **a** Neutrophil. **b** Platelets values. **c** Eosinophils. **d** Hemoglobin. **e** Interleukin-6

Neutrophil counts were significantly higher in the HDP group; and within this group they were significantly different in the three sub-groups in which we have classified them. The most marked differences were seen in the E and GH group when compared to N. Interestingly, neutrophil counts in PE were lower than in GH and significantly lower than in E. (Table 2: Fig. 1a).

**Table 2:** Hematological and cytokine parameters in HDP, in normotensive pregnant women and in non-pregnant women

Variable	Normotensive pregnant women (*n* = 77)	Hypertensive pregnant women (*n* = 76)	Non pregnant women [17]	*P* value
RBC count and RBC’s indices				
Mean RBC (SD)	3.85((0.41)	3.75 (0.66)	4.69 (3.84,5.59)	0.264
Mean Hb ± (SD)	10.99 (1.09)	10.06 (2.21)	13.5 (11.1,15.7)	0.001
Mean MCHC ± (SD)	32.06 (0.76)	31.77 (1.04)	32.7 (30.4,34.8)	0.051
Mean HCT ± (SD)	34.3 (3.25)	32.04 (5.6)	41.5 (36.2,46.8)	0.03
Mean MCV ± (SD)	89.04 (7.0)	86.00 (8.14)	89.5 (77.7–97.9)	0.014
Median MCH (IQR)	29.0 (26.7,30.6)	27.7 (24.9,29.6)	29.3 (24.2,33.1)	0.014
Median RDW (IQR)	16.45 (15.43,18.0)	18.1 (16.05,21.2)		< 0.001
White blood cell counts				
Median WBC count (IQR)	8.4 (7.0,10.3)	12.1 (8.9,15.05)	4.8 (3.2,8.0)	< 0.001
Median Neutrophil count (IQR)	5.6 (4.65,7.38)	9.7 (6.22,12.3)	2.3 (1.2,5.4)	< 0.001
Median Basophil (IQR)	0.04 (0.03,0.06)	0.04 (0.03,0.06)		0.924
Median Eosinophil count (IQR)	0.10 (0.5,0.2)	0.01 (0.003,0.05)		< 0.001
Median monocytes count (IQR)	0.55 (0.4,0.67)	0.53 (0.28,0.76)		0.462
Mean lymphocytes ± (SD)	1.90 (0.54)	1.73 (0.73)	1.9 (1.1,3.1)	0.124
Platelets counts and coagulation				
Median Platelets count (IQR)	232.5 (204,269)	196 (143,240)	271 (151,425)	< 0.001
Median PTT (IQR)	25.7 (24.6,26.6)	26.(24.5,27.5)		0.38
Median PT (IQR)	9.3 (9.1,9.8)	8.7 (8.2,9.5)		< 0.001
Median INR (IQR)	0.88 (0.85,0.92)	0.82 (0.78,0.88)		< 0.001
Cytokines				
Median interleukin 6 (IQR)	5.22 (4.8,6.3)	12.05 (6.31,23.91)		< 0.001
Median interleukin 10 (IQR)	42.4 (38.1,46.7)	46.7 (42.4,58.7)		< 0.001
Median TNF – α (IQR)	19.8 (17.5,23.4)	21.7 (17.9,26.1)		0.127

Platelet counts tended to be significantly lower in the entire HDP compared to N group. Although the difference between N and the GH group was not statistically significant, the differences were significant when comparing PE and E versus N (Table 2: Fig. 1b).

Compared to the normotensive group, the HDP group had significantly much lower eosinophil counts. Within the HDP group we observe a gradual decrease in median eosinophil count from GH to PE to E. (Table 2: Fig. 1c).

Differences in the distributions of Hemoglobin levels were marginal. However, taking all three hypertensive groups together the Hb level was significantly lower when compared to the normotensive group (Table 2: Fig. 1d).

Among the cytokines studied, IL-6 levels were significantly higher in the HDP group compared to the N group. Within the HDP group we observe a gradual increase in IL-6 from GH to PE to E (Table 2: Fig. 1e).

### Correlation of hematological parameters with cytokine levels

**Fig. 2:**
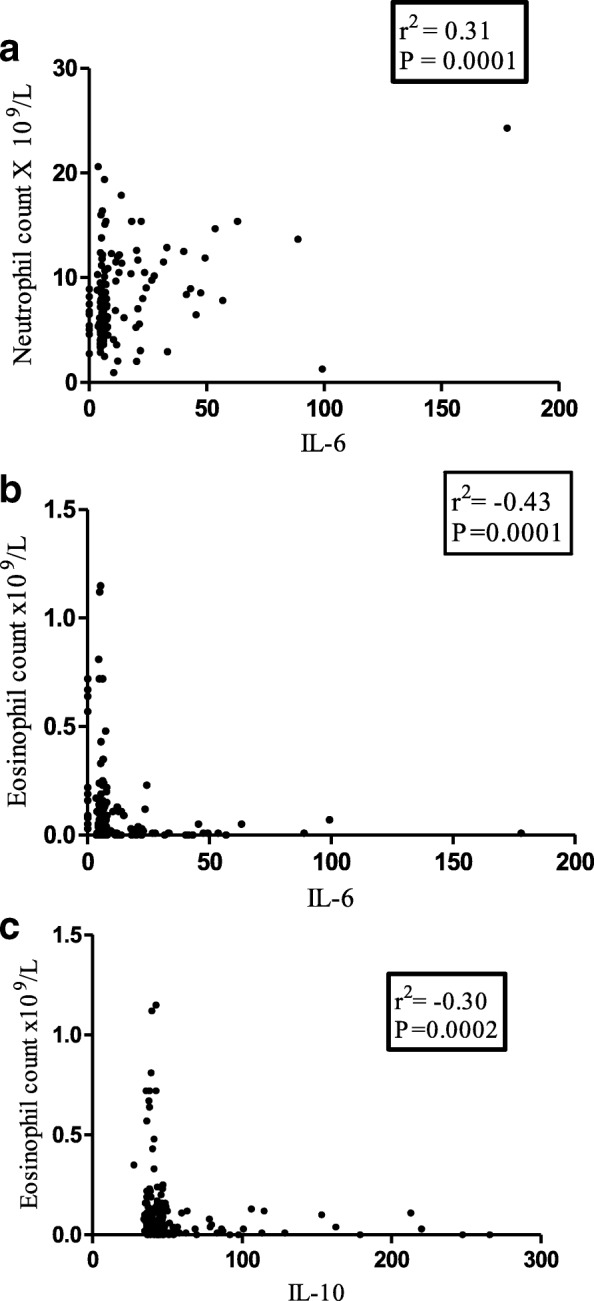
Several hematological parameters correlate with cytokine levels. **a**. Neutrophil count with IL-6 **b**. Eosinophil count with IL-6. **c.** Eosinophil count with IL-10

In order to determine the relationship between cytokines and hematological parameters during pregnancy Spearman’s rank order correlation was used. Significantly positive correlations were observed in IL-6 versus WBC count, IL-6 versus Neutrophil count and IL-6 versus lymphocytes count (Table 3). Conversely significantly negative correlation was observed in IL-6 versus Hb, IL-6 versus platelet count, IL-6 versus eosinophils count, IL-10 versus eosinophil count, TnF - α versus Hb, TNF - α versus MCH, TNF - α versus HCT and TNF - α versus MCV. (Table 3; Fig. 2).

**Table 3:** Cytokine levels correlate with hematological parameters in pregnant women (r^2^)

	Neutrophil counts	HB	Monocyte counts	Eosinophil counts	Platelet counts	MCH	HCT	MCV
IL-6	0.30^a^	−0.31^a^	0.18^a^	−0.43^a^	−0.22^a^	− 0.06	−0.29^a^	0.05
1 L-10	0.04	0.005	0.06	−0.30^a^	- 0.13	−0.02	−0.08	0.04
TNF	0.12	−0.22^a^	0.14	0.1	−0.1	−0.19^a^	− 0.20^a^	−0.18^a^

^a^Significant correlation

## Discussion

This study has revealed differences in hematological, coagulation and cytokine profiles in women with HDP compared to a normotensive control group. We have also detected significant correlations between cytokine parameters and hematological parameters.

### Anemia in pregnancy in relation to HDP

According to WHO definitions, any woman with a Hemoglobin level of less than 11 g/dl has anemia [18]. By this definition the frequency of anemia was 47.4% in our normotensive group, but 66.2% in our HDP group: however, in the majority of cases the anemia was mild (see Fig. 1a), and the differences between N and HDP were small. We observed a modest but significant negative correlation between the hemoglobin levels and IL-6 levels (Table 2; Fig. 2a). One possible explanation is that IL-6 may stimulate hepcidin production, with consequently reduced iron absorption in the duodenum [19]. However, the normal MCHC values in our patients do not suggest iron deficiency.

We also found a negative correlation between TNF alpha and hemoglobin levels. This might be explained directly by the fact that TNF alpha can inhibit erythropoiesis by favoring apoptosis [20].

The red cell indices MCV, MCH, MCHC were slightly lower in the HDP group than in the normotensive group (Table 2): this may be related to lower Hb levels, that have a negative correlation with TNF-α (Fig. 2b). The RDW was instead higher in HDP than in N (Table 2): in agreement with Kurt but not with Abdullah [21]. A high RDW is a quantitative expression of anisocytosis that may be associated with an inflammatory state (see below).

### An inflammatory state in normotensive and hypertensive pregnancy

Our finding that IL-10 and IL- 6 concentrations were significantly higher in the HDP group (Table 2; Fig. 1e) is in keeping with data systematically reviewed by Lau et al. [22]. In our study the interleukin 6 levels consistently increased with increasing severity of HDP, from GH to E. Our findings are essentially in agreement with those of others [8, 23–25] although there are some differences. Disparities in what has been observed in these studies might be attributed to many factors, such as diet and stress levels, which may directly or indirectly influence the production of these cytokines and which might differ in different study populations [26, 27].

As for the significance of increased levels of IL-6, we must consider that normal pregnancy entails a controlled inflammatory state [28], perhaps supported by cytokines produced by decidual cells and circulating mononuclear cells. When hypertension develops this control is disturbed, and the production of inflammatory cytokines increases [29]: this may trigger a cascade of events which are part of the pathophysiology of hypertensive disorders of pregnancy [12, 30].

Perhaps the most glaring expression of this inflammatory state is the leukocytosis of normotensive pregnant compared to non-pregnant women [17], and the specific increase in neutrophils in HDP [in agreement with Canzoneri et al. [31]].We suggest that increased neutrophil counts in HDP are a sign of inflammation, and they may be caused by increased IL-6 [32]: indeed, neutrophil counts correlated positively with IL-6 and IL-10 (Table 3).

### HDP is associated with eosinopenia

A finding we did not anticipate was that eosinophil counts were about 10-fold lower in HDP than in the normotensive group. Moreover, when we consider sub-groups within HDP we found that eosinophil counts became lower and lower with increasing severity from GH, to PE, to E (Table 2; Fig. 1c). These novel data may be attributed to increased production of glucocorticoids in HDP [33] that can cause eosinophil apoptosis [34]. At the same time, glucocorticoids increase production of IL-6 [33, 35]: this could underlie the negative correlation between IL-6 and Eosinophil that we have also observed (Fig. 2c).

The finding that IL-10 is negatively correlated with the eosinophil count does not support the notion that eosinophils produce IL-10. This negative correlation might result from a third factor which may again be the glucocorticoids, as they have been found to increase IL-10 [36].

### Thrombocytopenia in pregnancy is more prevalent in association with HDP

Thrombocytopenia is defined as platelet count of less than 150 × 10^9^/L [37]. By this definition the frequency of thrombocytopenia was 7% in our normotensive and 27% in our HDP mothers respectively: in the majority of cases thrombocytopenia was mild (see Fig. 1b). The difference between N and HDP was significant and in agreement with the studies by Freitas et al. and Onisai et al. [14, 38]. Low platelet counts in HDP have been suggested as a simple indicator for the risk of developing HDP and for the severity of HDP [11]. The observed low counts may result from platelet activation and subsequent consumption, that may be more pronounced in HDP [39].

### Coagulation

A hyper-coagulative or thrombophilic tendency is a physiologic feature of late stages of pregnancy, and it is more pronounced in HDP compared to normotensive: this has been attributed to increased thrombin generation in women with HDP [39, 40], Accordingly, we found that the median prothrombin time (PT) was lower in the HDP group than in the N group; for the partial thromboplastin time (PTT) the difference was not statistically significant (Table 2).

HDP, Inflammation and hypercoagulability both relate to endothelial dysfunction [41, 42], as this may result in activation of coagulation factors [43]. This in turn causes release of proteases that binds to the proteases activator receptors (PAR’s) [41], found on the surface of mononuclear cells, platelets and endothelial cells; PAR’s in turn will induce IL-6 and IL-8 production [44]. This may account for the modest but significant negative correlation between IL-6 and platelet counts observed in our study.

### Limitations

Our study was limited in that it was cross sectional: therefore, we are not reporting in this paper to what extent our findings will correlate with the clinical course of patients with hypertension in pregnancy. Also, we were not able to capture information on drug usage by study participants.

## Conclusion

We have found significant changes in hematological, cytokine and coagulation parameters in pregnant women with hypertensive disorders compared to normotensive pregnant women. The picture that emerges is that of an inflammatory state associated with hypertensive disorders of pregnancy. These changes are part of HDP, and there is a need to assess, in the context of sub Saharan Africa, how best we can use the hematological and cytokine profiles to monitor and control the HDP burden. Low dose Aspirin is a widely used measure to prevent HDP. Since aspirin is a powerful anti- inflammatory agent, its beneficial effect in this condition fits well with the notion that HDP is an inflammatory state. We believe that low dose aspirin should be standard of care in high risk pregnancies and in any pregnancy that develops HDP.

## Data Availability

Authors have had full control of all primary data, and they agree to share the data upon reasonable request.

## Abbreviations

BMI: Body mass index
EDTA: Ethylene-diamine-tetra-acetic Acid
GH: Gestational hypertension
Hb: Hemoglobin
HDP: Hypertensive Disorders of pregnancy
IL: Interleukin
INF: Interferon
INR: International Normalized Ratio
IUGR: Intrauterine Growth Retardation
MCH: Mean Cell Hemoglobin
MCHC: Mean Cell Hemoglobin concentration
MCV: Mean Cell Volume
MNH: Muhimbili National Hospital
MUHAS: Muhimbili University of Health and Allied Sciences
PAR: Protease activator receptors
PE: Pre-eclampsia
PLT: Platelet
PT: Prothrombin Time
PTT: Partial Thromboplastin Time
RDW: Red cell distribution width
SPSS: Statistical package for social sciences
TNF: Tumor necrosis factor
WBC: White Blood Cell
WHO: World Health Organization
